# Critiquing the Reasons for Making Artificial Moral Agents

**DOI:** 10.1007/s11948-018-0030-8

**Published:** 2018-02-19

**Authors:** Aimee van Wynsberghe, Scott Robbins

**Affiliations:** Technical University of Delft, Jaffalaan 5, 2628 BX Delft, Netherlands

**Keywords:** Artificial moral agents, Robot ethics, Machine ethics

## Abstract

Many industry leaders and academics from the field of machine ethics would have us believe that the inevitability of robots coming to have a larger role in our lives demands that robots be endowed with moral reasoning capabilities. Robots endowed in this way may be referred to as artificial moral agents (AMA). Reasons often given for developing AMAs are: the prevention of harm, the necessity for public trust, the prevention of immoral use, such machines are better moral reasoners than humans, and building these machines would lead to a better understanding of human morality. Although some scholars have challenged the very initiative to develop AMAs, what is currently missing from the debate is a closer examination of the reasons offered by machine ethicists to justify the development of AMAs. This closer examination is especially needed because of the amount of funding currently being allocated to the development of AMAs (from funders like Elon Musk) coupled with the amount of attention researchers and industry leaders receive in the media for their efforts in this direction. The stakes in this debate are high because moral robots would make demands on society; answers to a host of pending questions about what counts as an AMA and whether they are morally responsible for their behavior or not. This paper shifts the burden of proof back to the machine ethicists demanding that they give good reasons to build AMAs. The paper argues that until this is done, the development of commercially available AMAs should not proceed further.

## Introduction

Robots perform exceptionally well at clearly defined tasks like playing chess, assembling a car, classifying images, or vacuuming your floor. Increasingly, however, robots are being assigned more general tasks which require more than one skill. A driverless car, for example is supposed to get you from point A to point B following the rules of the road and reacting to unforeseen situations—like a child running out into the middle of the road after a ball. In order for robots to execute their function they require algorithms. These algorithms controlling robots are becoming increasingly autonomous and often require artificial intelligence (AI). As autonomy in robots and AI increases so does the likelihood that they encounter situations which are *morally salient*. As of 2017 robots are and will continue to be designed, developed and deployed in morally salient contexts; from robots in the hospital lifting and/or bathing patients to robots in the military assisting with bomb disposal or intelligence gathering.

The Executive Summary of the International Federation of Robotics^1^ shows a marked increase in robot sales across every sector from 1 year to the next; including a 25% increase in the total number of service robots sold in 2015 alone. These robots can be used to save lives, to assist in dangerous activities, and/or to enhance the proficiency of human workers. Many industry leaders and academics from the field of machine ethics—the study of endowing machines with ethical reasoning—would have us believe that robots in these and other morally charged contexts will inevitably demand that these machines be endowed with moral reasoning capabilities. Such robots are often referred to as artificial moral agents (AMAs). In this paper the variety of reasons offered by machine ethicists in favor of AMAs are challenged. This paper asks: are the given reasons adequate justification for the design and development of AMAs?

From the academic domain a variety of scholars in the fields of ethics and technology and/or robot ethics have argued against the development of AMAs (Bryson 2008; Johnson and Miller 2008; Sharkey 2017; Tonkens 2009). What is currently missing from the debate on AMAs is a closer look at the reasons offered (to society, academics, the media) by machine ethicists to justify the development of AMAs. This closer inspection is particularly compulsory given the amount of funding allocated to the development of AMAs (from funders like Elon Musk) coupled with the amount of attention researchers and industry leaders receive in the media for their efforts in this direction.^2^ Moreover, the stakes are high because the resulting technology could create novel demands on society; questions about what counts as an AMA, whether they are deserving of citizenship,^3^ and/or whether they are morally responsible for their behavior or not. In other words, a machine with moral reasoning capabilities might be thought to deserve moral consideration in the form of rights and/or protections (Coeckelbergh 2010; Darling 2012; Gunkel 2014).

In order to examine the justifications for AMAs, this paper begins with a description of the field of machine ethics: what it is, the terminology used, and the response to machine ethics found in the literature by robot ethicists and scholars in the field of ethics and technology. In subsequent sections, the reasons offered in favor of developing robots with moral reasoning capabilities are evaluated. It is argued that each of the reasons lack both empirical and intuitive support. The burden of proof is thereby shifted to machine ethicists to justify their pursuits.

## Machine Ethics

Summarized by machine ethicist Susan Anderson, the “ultimate goal of machine ethics is to create autonomous ethical machines” (2007, p. 15). The term machine ethics was first used in 1987 by Mitchell Waldrop in the AI Magazine article “A Question of Responsibility” (Waldrop 1987). In 2005 the AAAI held a symposium on machine ethics which resulted in the edited volume *Machine Ethics* in 2011 by Susan Leigh and Michael Anderson (Anderson and Anderson 2011). The field may be referred to by other names, e.g. machine morality, but for the purposes of this paper, machine ethics is a field of study dedicated to the computational entity as a moral entity.^4^

There are several phrases and terms for discussing robots with moral reasoning capabilities (e.g. moral machines, implicit vs explicit ethical agents).^5^ For the purposes of this article, however, the term artificial moral agent (AMA) will be used for consistency and clarity.^6^ This clearly restricts the discussion to robots capable of engaging in autonomous moral reasoning, that is, moral reasoning about a situation without the direct real time input from a human user. This moral reasoning is aimed at going beyond safety and security decisions about a context. How this might be done, and whether or not this can be achieved in practice, are questions that go beyond the scope of this paper (these are the questions underpinning the field of machine ethics itself). Rather, the interest of this paper is in targeting the reasons offered in support of developing such machines.

What a robot or machine would act like if it were to think in an ethical way is a central feature in the 1950 works of science fiction writer Isaac Asimov. Asimov, who coined the term ‘robotics’ (the study of robots) is best known for his work articulating and exploring the three laws of robotics (Asimov 1963). In short, these three laws were a kind of principled or deontological approach to embedding ethics into a machine. Through a series of short stories Asimov reveals the difficulty and nuances of robots acting in an ethical manner because each ethical principle conflicts with another to such a degree that experience, wisdom, and intuition are required to come to a solution or resolution of the conflict. His stories highlight the struggle to define ethics in a computational form.

From the academic domain a variety of scholars in the fields of ethics and technology and/or robot ethics have argued against the development of AMAs. On one hand, scholars insist that the technology ought to be designed in such a way that responsibility distribution remains “tethered to humans” (Johnson and Miller 2008). Similarly, computer scientist Joanna Bryson argues that robots ought to remain in the instrumental service of humans, as slaves if you will, meeting the needs of their human users and intentionally designed not to be a moral agent (Bryson 2008). This claim is predicated on the assumption that humans will own robots and as such will be responsible for their existence and capacities. On the other hand, philosopher Ryan Tonkens argues that given the impossibility of finding universal agreement concerning the ethical theory used to program a machine, the initiative is moot (Tonkens 2009).

Outside of these arguments robot ethicist Amanda Sharkey outlines the misappropriation of the use of ‘ethical’ in the quest to make moral machines and insists on the creation of “safe” machines instead. In the same line of thinking, Miller et al. argue that responsible development requires careful use of terminology and representation in the media (Miller et al. 2017).

The above arguments are still waiting to be adequately answered by the machine ethics community. However, the purpose of this paper is to question the positive reasons offered by the machine ethicists *for* building AMAs. These reasons have not yet been fully evaluated as yet and a closer inspection of them reveals a lack of sufficient justification. Given the high stakes and of the research and development in question coupled with the current speed of (and funding for) machine ethics initiatives these must be addressed now.

## Reasons for Developing Moral Machines

Machine ethicists have offered six reasons (found in the literature) in favor of and/or promoting the development of moral machines. These are not stand alone reasons; rather, they are often intertwined. Part of the reason it sounds so convincing (at first glance) is because of their interdependency rather than the strength of any reason on its own. Disentangling these reasons shows their dubious foundation and allows one to challenge the endeavor of machine ethics.

### Inevitability

Robots with moral decision making abilities will become a technological necessity (Wallach 2007).

[Artificial Moral Agents] are necessary and, in a weak sense, inevitable (Colin Allen and Wallach 2011).

Machine ethicists claim that robots in morally salient contexts will not and cannot be avoided, i.e. their development is inevitable (Anderson and Anderson 2010; Moor 2006; Scheutz 2016; Wallach 2010).

First, what exactly is meant by morally salient contexts is unclear. For some researchers this would include contexts such as healthcare, elder care, childcare, sex, and or the military—where life and death decisions are being made on a daily (or hourly) basis (Arkin 2009; Lokhorst and van den Hoven 2011; Sharkey 2016; Sharkey 2008; Sharkey and Sharkey 2011; Sharkey et al. 2017; van Wynsberghe 2012). There is no question that robots are entering these service sectors. The International Federation for Robotics Executive Summary of 2016 tells us that “the total number of professional service robots sold in 2015 rose considerably by 25% to 41,060 units up from 32,939 in 2014” and “service robots in defense applications accounted for 27% of the total number of service robots for professional use sold in 2015”. Moreover, sales of medical robots increased by 7% from 2014 to 2015.^7^

For others, morally salient context is much broader than a pre-defined space or institution:

any ordinary decision-making situation from daily life can be turned into a morally charged decision-making situation, where the artificial agent finds itself presented with a moral dilemma where any choice of action (or inaction) can potentially cause harm to other agents. (Scheutz, 2016, p. 516)

From the above quote Scheutz is saying that a morally charged situation can arise at any moment in the event that someone could be harmed through (in)action of a robot. This thin description of a morally charged decision making situation adds further ambiguity to the discussion, namely (1) what level of autonomy does the robot have, and (2) what definition of harm is Scheutz talking about? There seems to be an assumption being made in the above quote concerning the robot that the robot must make a choice for action or inaction and thus that the robot must be autonomous. According to Scheutz then any autonomous robot interacting with a human user that has the potential to harm its user should be endowed with moral reasoning capabilities. What would Scheutz have us do with industrial robots that possess divergent levels of autonomy, work with humans in their presence, and for which it has already been shown that the robots can bring serious harm or sometimes death, to humans? Scheutz’s position would imply that industrial robots as well ought to be developed into AMAs.

Consider also the definition of ‘harm’ that ought to be adopted. Is it only physical harm to the corporeal body and mind that is the object of discussion here and if so what about the robot’s, or AI algorithm’s, ability to collect, store and share information about its users in a home setting? Considering the real possibility that home robots will be connected to the Internet of Things (IoT) which holds the potential for hackers and/or for companies not related to the robotics company to access personal data from users. The harm that can come from the mis-appropriation of one’s data has proven to be noteworthy of late: people can be refused mortgage loans, stalked, blackmailed, harassed online, or worse. If harm is to be extended to include the risk of one’s digital information, and interaction with a machine that might cause harm demands that it be endowed with ethical reasoning capacities, then one must concede that every device that one interacts with in a day (your tv, phone, fridge, alarm clock, kettle, etc.) ought to have such capabilities. Thus, Scheutz’s position leads to the conclusion that any technology that one interacts with and for which there is a potential for harm (physical or otherwise) must be developed as an AMA and this is simply untenable.

Second, a distinction must be made between *being in a morally charged situation*, on the one hand, and *being delegated a moral role* on the other. Consider animals used for therapeutic purposes in an elderly care facility; one would never demand that a dog placed in this context would need to reason ethically because of its role in therapy and the potential for harm in this context. Indeed the dog would be trained to ensure a degree of safety and reliability when interacting with it but would the dog be a *moral dog* in the end?^8^

With this thought in mind let us day the discussion will be limited to an examination of a *morally salient context* to contexts such as the military and healthcare, which are often thought of as morally salient, and agree that it is inevitable that robots will be placed within these contexts. In this case there is a different, more nuanced problem that can be put into the form of a dilemma: when placed in a morally salient context either machines will be delegated a moral role or they will not. If one chooses the first horn—that the machine is delegated a moral role—then one must accept that it is inevitable that machines will be delegated a moral role in addition to the inevitability of the machine being in this morally salient context. However, this is simply not the case. There are plenty of machines operating in morally salient contexts which have not been delegated a moral role and are providing a valuable service. Consider for example:

Corti is an AI which listens in on emergency phone calls and makes correlations between the breathing and speech patterns of a caller and the risk of heart attack (Peters 2018). The information from the AI is presented to the phone operator to assist in their decision making. In short, the AI is a support system for the decision making of the operator. This may be akin to the cancer detection AI or other more traditional technologies in the operating suite (e.g. electrocardiogram, respiratory monitor and so on). Corti is clearly in a morally salient context (life and death), yet the machine is not delegated a moral role. The human being is still in charge and holds all of the responsibility for decision making. If one agrees with machine ethicists then one should accept that it is inevitable that a moral role reserved for the human in this case will be assigned to the machine. While this is probably unnecessary and most likely harmful, the point is that there is simply no reason to believe that this is *inevitable*.

If, however, one takes the other horn of the dilemma then the claim is as follows: robots will inevitably be in morally salient contexts without being delegated a morally salient role. The problem with this is that there is little new here. Microwaves and coffee machines exist in the hospital with no need for moral reasoning capabilities; this horn should be of little interest to machine ethicists. In short, there is not any evidence to suggest that it is inevitable that there will be a need for machines with moral reasoning capabilities regardless of whether or not they function in a morally salient context.

### Artificial Moral Machines to Prevent Harm to Humans

For many scholars the development of moral machines is aimed at preventing a robot from hurting human beings. To ensure that humans can overcome the potential for physical harm, a technological solution is presented; namely, to develop AMAs:

the only way to minimize human harm is to build morally competent robots that can detect and resolve morally charged situations in human-like ways (Scheutz 2016).

The line of reasoning here is pretty straight forward in that: “it is clear that machines…will be capable of causing harm to human beings” (Anderson and Anderson 2010) and this can be mitigated and/or reduced by endowing the robot with ethical reasoning capabilities. This also speaks to the interconnection of the reasons in favor of AMAs; robots are inevitable, robots could harm us, therefore robots should be made into AMAs.

It is unclear that AMAs are the solution to this problem, however. There are plenty of technologies capable of harming human beings (e.g. lawn mowers, automatic doors, curling irons, blenders); the solution has always been either to design them with safety features or to limit the contexts in which a technology can be used. An elevator door has a sensor so that it does not close on people; lawn mowers have a guard to protect us from their blades; and ovens have lights to warn us when our stovetop is hot. One does not normally use barbeques indoors or chainsaws in daycare centers. Machine ethicists are the first to suggest endowing technology with moral reasoning capabilities as a solution to problems of safety.

Furthermore, machine ethicists may also agree with the pursuit of safe robots and then the real concern for ethicists is that ethics is being reduced to safety. Notions such as values, rights, freedoms, good vs bad, right vs wrong, are central to the study of ethics and form the basis for a discussion of competing conceptions of the good life. One may believe that the values of safety and security are fundamental to achieving the good life; however, ethics cannot be reduced to these issues. So if AMAs are simply a solution to possibly harmful machines, then *safety—*not *moral agency—*is the object of debate.

In this case the world ‘moral’ is a linguistic ‘trojan horse’—a word

that smuggles in a rich interconnected web of human concepts that are not part of a computer system or how it operates (Sharkey 2012, p. 793)

The concept of *moral* machines or artificial *moral* agents invites, or more strongly requests that, the user believe the robot may care for him/her, or that the robot can experience feelings. For robot developers this could increase desirability of the robot and therefore profits. However, this is problematic for the public in that it invites a kind of fictive, asymmetric, deceptive relationship between human and robot.

Thus, machine ethicists must either distinguish what makes their machines “moral” above and beyond “safe” or they must stop using the world “moral” as the word is not appropriate—only the most reductionist account of morality would equate it with preventing harm.

### Complexity

as systems get more sophisticated and their ability to function autonomously in different contexts and environments expands, it will become more important for them to have ‘ethical subroutines’ of their own (Allen et al. 2006, p. 14)

The idea behind using complexity as an argument in favor of AMAs is that robots are, and will increasingly become, so complex in terms of their programming that it is no longer possible know what they will do in novel situations. This uncertainty results in the impossibility of the engineer to predict every scenario and as such it will not be possible for the engineer to predict the robot’s actions. Consequently, one cannot simply foresee a morally problematic situation and pre-program what the robot should do. Instead, authors who use the complexity argument to promote development of AMAs claim the robot needs to have moral competence in order to govern its unpredictable actions in the inevitably unpredictable and unstructured human environments that the robot will be placed.

First, using complexity as a reason for developing AMAs expects both that there will be complex robots and that such robots ought to be placed in contexts for which this complexity (i.e. unpredictability) could cause problems for human beings.

Next, of importance for this issue is the context within which the robot will be placed. In other words, this problem can be mitigated simply by restricting the context within which these machines are used. For example, designers of Google’s complex machine AlphaGo may not have any idea what their machine will do next (which move it will make in the notoriously difficult game of GO); however, this is not an ethical or moral problem because the context (the game of GO) is restricted. Its complexity will not pose a problem for us.

One may argue that human beings are unpredictable and can cause harm to other human beings. The solution has not been to prevent the delegation of moral roles to human beings. One might ask: why treat machines differently? While it is outside of the scope of this paper to engage in a debate on just how predictable humans are, it can be noted that with regard to serious moral values—killing, non-consensual sex, harming innocent people for fun—society places restrictions on unpredictable human beings (i.e. imprisonment). Humans may be unpredictable in terms of what they will do next, but most of us assume that a random person will not intentionally cause us harm.

### Public Trust

Other machine ethicists argue that making AMAs will increase public trust: “Constructing artificial moral agents serves at least two purposes: one, better understanding of moral reasoning, and, two, increasing our trust and confidence in creating autonomous agents acting on our behalf” (Wiegel 2006). There has been talk in the media expressing concerns surrounding AI and robotics—voiced by the likes of Elon Musk and Steven Hawking (Cellan-Jones 2014; Markoff 2015). Rather than preventing the development of robots that are the source of these fears “machine ethics may offer a viable, more realistic solution” (Anderson and Anderson 2007).

This line of thinking assumes that if robots are given moral competence then this will put the public at ease and lead to public acceptance. It should be noted here that acceptance differs from acceptability. As an example, the public may *accept* geo tagging and tracking algorithms on their smartphone devices but this does not meant that such privacy breaching technologies and/or the lack of transparency about their existence are *acceptable* practices for upholding societal values.

Some important clarifications are needed when discussing trust as a concept. Traditionally speaking, trust is described as an interaction between persons or between a person and an institution and so on. For scholar John Hardwig trust can be placed in people, processes and in knowledge (Hardwig 1991). In more recent years scholars are discussing a new form of trust; trust in algorithms (Simon 2010). This new form of trust is most commonly referred to as ‘algorithmic authority’ and is described as a practice of placing confidence in the decisions made by an algorithm (Shirky 2009). Wikipedia is an example of this form of trust as it requires trust not in persons but in the algorithms regulating the content on the website.^9^

If trust is broken the result will be feelings of disappointment on the part of the truster. These resulting negative feelings are what relate trust to the concept of reliability: if either one is misplaced the result is oftentimes feelings of disappointment (Simon 2010). Trust is distinguished from reliability in the intensity of the emotions experienced afterwards; “trust differs from reliance because if we are let down we feel betrayed and not just disappointed” (Baier 1986; Simon 2010). Relatedly, Simon claims that one cannot speak of trust for socio-technical systems but rather of reliance: “we usually do not ascribe intentionality to unanimated objects, which is why we do not feel betrayed by them” (p 347). Hence, we do not trust unanimated objects, we rely on them.

With the formulation of Hardwig in mind—trust can be placed in people, processes, and in knowledge and when it concerns placing trust in robots one must ask: who, or what, are machine ethicists asking the public to trust: the algorithm directing the robot; the designer; or, the development process?

If the public is being asked to trust the algorithm then one must consider that:

unfortunately we often trust^10^ algorithms blindly. Algorithms are hidden within a system. In most cases we are not aware of how they work and we cannot assess their impact on the information we receive. In other words: algorithms are black-boxed (Simon 2010).

Consequently, if the public is being asked to trust an algorithm and it is considered a black-box, then, as Simon rightly asserts, it must be *opened*—the way it works, the decisions made in its development, and alternatives—must be made transparent and subject to scrutiny.

If, however, the public is being asked to trust the designer then designers and developers ought to develop a code of conduct (perhaps in the form of soft law) to adhere to. Again, transparency of this is required for the public to have the knowledge required for trust.

Last, if the public is being asked to trust the process through which the robot is being developed, a kind of procedural trust, then standards and certifications must be developed to once again provide the user with the knowledge required to place trust in the process through which the robot was developed. Examples of such procedural trust are FairTrade, ISO, GMOs, and so on.

In any case it is important to point out the inconsistency between the promotion of AMAs for reasons of complexity and for reasons of trust: it is inconsistent to expect unpredictability in a machine and to expect trust in a machine at the same time. While this may not be the case for people—one might trust persons who are at the same time unpredictable—more clarity is needed in understanding who/what society is being asked to trust and what level of (un)predictability one can assume.

### Preventing Immoral Use

In the 2012 American science fiction comedy-drama movie “Robot & Frank” there is a compelling story of how a retired cat burglar convinces his robot to help him enter the business once again. The story raises the question about human–robot interaction not in the sense of safe or reliable interactions but rather should the robot be capable of evaluating a human’s request for action. Thus, another reason put forward for the development of AMAs can be stated as: preventing humans from misusing, or inappropriately using, a robot requires that the robot be developed as a moral machine and can thus prevent misuse of itself, itself.

The main problem with this reason has to do with the potential to constrain the autonomy of humans. It’s not always clear what is the good thing to do and oftentimes context is required for this (Miller et al. 2017). Consider for example a couple who are at home having a few drinks together, domestic violence ensues after a heated argument, the women tries to get away in her car but the breathalyzer in her car picks up the alcohol in her system and the car won’t start. What would be the right thing to do in such an instance? How should the device be programmed? A deontologist would have us believe that one should never get behind the wheel after consuming alcohol, while a utilitarian might suggest that if the woman’s life is saved in doing so the overall good is maximized (unless of course the women were to get into a car accident and harm two others). These are not easy problems to solve and require particular details of context and individuals.

Consider another example where misuse is unclear. If an elderly person at home wants to have a fourth glass of wine and asks his/her robot to fetch it. If the robot fetches the wine, is the robot being misused in so far as it is contributing to poor health choices of the user? Or is the robot ‘good’ in so far as it fulfilled the request of its user. Presenting scenarios like these is meant to show the difficulty in determining the right or the good thing to do. And yet if one is claiming that robots should be involved in the decision making procedure it must be very clear how a ‘good’ robot is distinguished from a ‘bad’ one.

### Morality: Better with Moral Machines

Endowing the robot with the capability to override or edit a human’s decisions draws us into the discussion of the robot as a superior moral reasoner to a human. Computer science Professor James Gips suggested back in 1994 that “not many human beings live their lives flawlessly as moral saints. But a robot could (Gips 1994, p. 250). Also along the same lines, Professor of Philosophy Eric Dietrich has suggested that:

humans are genetically hardwired to be immoral…let us – the humans – exit the stage, leaving behind a planet populated with machines who, although not perfect angels, will nevertheless be a vast improvement over us (Dietrich 2001)

The assumption here is that a robot could be better at moral decision making than a human given that it would be impartial, unemotional, consistent, and rational every time it made a decision. Thus, no decisions would be based on bias or emotions, no decision would be the result of an affinity towards one person (or group of people) over another. More importantly, the robot would never tire but would have the energy to be consistent in decision making: to make the same choice time after time.

This line of reasoning to promote AMAs is also often invoked when speaking of robots in military contexts. In particular computer scientist/roboticist Ronald Arkin discusses the power of autonomous military robots for overcoming the shortcomings of humans on the battlefield (Arkin 2009). These robots would not rape or pillage the villages taken over during wartime and would be programmed as ethical agents according to the Laws of Just war and/or the Rules of Engagement.

There are some general concerns with this reason. First, the underlying programming which will enable machines to reason morally implies that one has an understanding of moral epistemology such that one can program machines to “learn” the correct moral truths—or at least know enough to have AMAs learn something that works. This gets complicated as there is no moral epistemology which does not have serious philosophical objections and therefore presents a barrier to being reduced to a programming language.

Machines could only be better if there is some standard of moral truth with which to judge. This implies that there are objective moral truths in a moral realist sense and further that it is possible to know what they are. This is opposed to error theory (the idea that there are no moral truths at all—so nothing to know), and moral skepticism (there are moral truths, but it is not possible that we as humans can know them).

Furthermore, based on the above quotes it seems that the moral truths that machines would be better at knowing are truths which are independent of human attitudes. Russ Shafer-Laundau calls these stance-independent moral truths (Shafer-Landau 1994). If—and that is a big if—there are stance independent moral truths whereby the truths have no dependence upon human desires, beliefs, needs, etc. then there are objections to how one could come to know such truths (Finlay 2007). If a machine was built which did somehow discover moral truths that have heretofore yet to be discovered (because morality would be a lot easier if we simply knew the moral truths) then one would have to accept on faith that machines are better than we are.

The moral consistency promised by machine ethicists is only a public good if the moral truths are known in advance—the opposite of the situation human beings find themselves in. For, as shown in previous sections, AMAs are argued to be needed because one cannot predict the kind of situations or moral dilemmas they will be faced with. But this is not a chess game where the outcome is a win or a loss. An autonomous car which drives off a cliff—killing its one passenger—in order to save five passengers in another car would not be a clear cut situation that everyone could agree was the correct decision. Indeed, books are written about that very decision and human disagreement about what should be done (i.e. the trolley problem) (see e.g. Greene 2013).

Lastly this all presumes that human emotions, human desires, and our evolutionary history are all getting in the way of our moral reasoning—causing it to be worse than it could be. There are those who include moral emotions as a necessary part of moral judgment and reasoning (Kristjánsson 2006; Pizarro 2000; Roeser 2010). If this is so, then AMAs would require emotions—something not even on the horizon of AI and robotics.

Let us say that there are moral principles and that humans can know what they are. So there is a standard with which to judge AMAs. Furthermore, let us also assume they live up to their promise and are better moral reasoners than humans. It might then make sense to outsource our moral decisions to machines. This would assume that being good at moral reasoning is not a necessary part of a human being’s good life. Aristotle believed leading a moral life and gaining a moral understanding through practice was necessary to leading a good life (Aristotle et al. 1998). Many contemporary philosophers agree. Outsourcing our moral reasoning to machines could cause an undesirable moral deskilling in human beings (Vallor 2015). The point is that it is not clear at all if machines were better moral reasoners than us that this would be a good reason to use them. Added to this, to make such an assumption is to assume we have an understanding of morality and the good life that we may not.

### Better Understanding of Morality

Finally, machine ethicists sometimes argue that developing robots with moral reasoning capabilities will ultimately lead to a better understanding of human morality:

the hope is that as we try to implement ethical systems on the computer we will learn much more about the knowledge and assumptions built into the ethical theories themselves. That as we build the artificial ethical reasoning systems we will learn how to behave more ethically ourselves (Gips 1994)

In short, regardless of the resulting machine the very process of attempting to create such a machine would benefit humans in so far as we would learn about ourselves and our moral attributes (Gips 1994; Moor 2006; Wiegel 2006).

The most important consideration in response to this claim is that ethical theories are not (and have little to do with) how people reason morally so the work doesn’t help understand *human* morality. Experiments in moral psychology show us that human morality is deeply influenced by irrelevant situational factors (Doris 1998; Merritt 2000), is driven by emotion (Haidt 2001; Haidt and Joseph 2008), and influenced by our evolutionary past (Street 2006). To be sure, there is an intense debate in the literature with regard to each of these studies. The point is that human morality, in the descriptive sense, is dependent upon many complex factors and building a machine that tries to perfectly emulate human morality must use each of these factors combined rather than rely on ethical theory alone.

## Conclusion

In this paper the reasons offered by machine ethicists promoting the development of moral machines are shown to fall short when one takes a closer look at the assumptions underpinning their claims and/or the claims themselves. While autonomous robots and AI can and should be used in morally salient contexts this need not require that the robot be endowed with ethical reasoning capabilities. Merely placing something in an ethical situation, like a heart monitor in an ICU hospital ward, does not also demand the thing to ethically reflect on its course of action. The power of such robots in said contexts can still be harnessed even without making them into so-called moral machines.

This article has shown here that AMAs are promoted for reasons of inevitability, complexity, establishing public trust, preventing immoral use, because they would be better moral reasoners than us, or because there would be a better understanding of human morality with AMAs. None of these reasons—as they have been articulated in the literature—warrant the development of moral machines nor will they work in practice. This is so because of: inherent bias to learn how to be ethical, the impossibility or difficulty of understanding the complexity of the robot’s decision, how to evaluate or trust the superior ethical reasoning of the robot and so on.

There are dangers in the language used for these endeavors. One should not refer to moral machines, artificial moral agents, or ethical agents if the goal is really to create safe, reliable machines. Rather, they should be called what they are: safe robots. The best way to avoid this confusion, considering that no critical or unique operational function appears to be gained through the endowment of ethical reasoning capabilities into robots, is to simply not do it. To that end the authors suggest an implication for policy makers and academics: place a moratorium on the commercialization of robots claiming to have ethical reasoning skills. This would allow academics to study the issues while at the same time protecting users—the consumer, the indirect user, and society at large—from exposure to this technology which poses an existential challenge.

In closing, our goal for this paper was to pick apart the reasons in favor of moral machines as a way of shifting the burden of proof back to the machine ethicists. It is not up to ethicists anymore to tell you why they think the pursuit of an AMA is flawed; rather, now that it has been shown that the motivations for developing moral machines do not withstand closer inspection machine ethicists need to provide better reasons. So, to the machine ethicists out there: the ball is in your court.

## Footnotes

1 For more on this see https://ifr.org/ifr-press-releases/news/world-robotics-report-2016.

2 For more on the popular news articles see: (Deng 2015; “Morals and the machine” 2012; Rutkin 2014).

3 Robot Sophie of Hanson Robotics, first robot granted citizenship in Saudi Arabia, see (Gershgorn 2017; Hatmaker 2017).

4 For more readings on machine ethics see Wallach and Allen (2010), Anderson and Anderson (2007, 2011), Anderson (2011), Moor (2009, 2006), Scheutz (2016), and Allan et al. (2006).

5 For more on this see Wallach and Allen (2010), Moor (2009, 2006).

6 The concept and notion of artificial moral agents has built momentum as a thought experiment and/or a possible reality. For a rich and detailed discussion of AMAs the authors recommend the following: (Allen et al. 2005, 2000; Floridi and Sanders 2004; Himma 2009; Johnson and Miller 2008; Nagenborg 2007; Wiegel 2010).

7 For more on this please refer to: https://ifr.org/downloads/press/02_2016/Executive_Summary_Service_Robots_2016.pdf.

8 See also the work of van Wynsberghe illustrating how robots in healthcare need not be delegated roles for which ethical reasoning and/or moral responsibility are required. (van Wynsberghe 2012, 2013, 2015, 2016). Furthermore there are existing frameworks and applications for realizing ethical values in technological design. See e.g. (Friedman and Nissenbaum 1996; Nissenbaum 2001; van de Poel 2013; van den Hoven 2007; van Wynsberghe and Robbins 2013).

9 This form of trust may also be referred to as procedural trust (Simon) as it concerns trust in the process through which knowledge is created rather than in actions of persons.

10 The word trust is used here because it comes from a quotation; however, it should be noted that the authors are inclined to use the work rely instead.
